# The impact of calorific screening thresholds and weight status when validating UK supermarket transaction records in dietary evaluation: FIO-STRIDE

**DOI:** 10.1038/s41366-026-02107-1

**Published:** 2026-05-25

**Authors:** Thomas Sawczuk, Hannah C. Greatwood, Mark S. Gilthorpe, Michelle A. Morris, Victoria Jenneson, Emma Wilkins, Mark A. Green, Alexandra M. Johnstone, Claire Griffiths

**Affiliations:** 1 Obesity Institute, School of Sport, Leeds Beckett University, Leeds, UK; 2 Carnegie Applied Rugby Research (CARR) Centre, Carnegie School of Sport, LBU, Leeds, UK; 3 School of Food Science and Nutrition, University of Leeds, Leeds, UK; 4 Leeds Institute for Data Analytics, University of Leeds, Leeds, UK; 5 School of Geography, University of Leeds, Leeds, UK; 6 Department of Geography and Planning, University of Liverpool, Liverpool, UK; 7 Rowett Institute, School of Medicine, Medical Sciences and Nutrition, University of Aberdeen, Aberdeen, UK

**Keywords:** Weight management, Nutrition, Public health

## Abstract

**Background:**

Supermarket transaction data has the potential to provide a wide-scale understanding of population dietary behaviours, but its relationship with consumption is unclear as purchases are typically made at the household level while consumption occurs at the individual level. These behaviours may differ by weight status. This study assessed whether calorific screening thresholds improved the agreement between objective consumer purchase data and self-reported dietary intake for people living with (PLWOw/Ob_with_) and without (PLWOw/Ob_without_) overweight/obesity.

**Methods:**

Participants (*N* = 642) from a retailer’s loyalty card database completed a 170-item food frequency questionnaire, shared transaction records, height, weight, and household composition data for this study. Nutrients (energy, sugar, total fat, saturated fat, protein, and sodium) from supermarket transaction purchases were allocated to the study individual proportionally based on their household composition. Bland-Altman analyses were used to assess the agreement and bias between objective consumer purchase data and self-reported dietary intake globally, and between PLWOw/Ob_with_ and PLWOw/Ob_without_, across a range of calorific screening thresholds.

**Results:**

No agreement was identified between objective consumer purchase data and self-reported dietary intake for any of the nutrients when the data were analysed without screening thresholds. However, agreement was identified when screening thresholds were employed at ≥1000 Kcal/day (energy, sugar, total fat and saturated fat) or ≥1500 Kcal/day (protein and sodium). PLWOw/Ob_with_ consumed greater energy (19%), sugar (36%), total fat (22%) and saturated fat (25%) than they were estimated to have purchased at the retailer. PLWOw/Ob_without_ only consumed more sugar (19%).

**Conclusions:**

The application of screening thresholds based on estimated individual calories purchased may provide a valuable preprocessing step within the analysis of consumer purchase data, allowing agreement to be achieved for absolute nutrient values. Differences in bias between PLWOw/Ob_with_ and PLWOw/Ob_without_ show that insights into purchase and consumption patterns can be identified using consumer purchase data.

## Introduction

Unhealthy dietary behaviours are major contributors to the global burden of disease, accounting for substantial disability-adjusted life years [1]. Assessment of individual dietary behaviour often relies on self-reported methods, such as Food Frequency Questionnaires (FFQs), to estimate individual consumption patterns [2]. These assessment methods are prone to recall bias and inaccuracies, particularly when respondents misreport portion sizes or frequency of consumption [3]. Consequently, alternative data sources, such as supermarket transaction records, which represent the upstream dietary behaviours that occur before consumption, have been considered as proxies for estimating nutrient intake [4, 5]. However, previous research has demonstrated mixed results when comparing self-reported and transaction-based dietary assessments [6, 7].

The relationship between supermarket transaction data and food consumption is complicated since not all food purchased at a supermarket is consumed by the purchaser. Challenges include consumption occurring at the individual level while supermarket transactions are recorded at the household level, the contribution of out-of-home foods, and inconsistent shopping habits [8, 9]. Previous research has incorporated the allocation of household food purchases to the individual level [4], but inconsistent shopping habits (e.g. shopping at different retailers at different times or for different foods, loyalty cards not being scanned for every purchase, or multiple individuals within the same household using different cards for their purchases) are yet to be considered. These habits cause problems when evaluating the agreement between purchase and consumption data as participants may be included in studies when their supermarket transaction data could not possibly represent their individual consumption. For example, a healthy participant who purchases ~500 Kcal/day from a single retailer will never show agreement between their transaction purchases from the retailer and their individual consumption in absolute terms as they are likely to consume ~2000–2500 Kcal/day based on nutritional recommendations [10], so the remaining consumed energy must have been purchased elsewhere. Previously, a self-reported higher percentage of purchases from a retailer has been associated with greater correlations between self-reported food purchase and consumption data [6], but these data are not objective, nor are they readily available. Therefore, it would be useful to assess whether a calorific screening threshold could be used to identify study participants who purchase a substantial quantity of food from the retailer relative to their total dietary intake within a study period. Including only these participants might enable stronger agreement to be found between supermarket transaction data and individual consumption, increasing confidence in the findings from consumer purchase data studies.

It has been suggested that people living with obesity (PLWOw/Ob_with_) are more likely to underreport dietary intake compared to those living without (PLWOw/Ob_without_) [11, 12]. However, it is currently unclear how this is reflected in food purchases from supermarkets. For example, it is currently unknown whether PLWOw/Ob_with_ or PLWOw/Ob_without_ purchase greater quantities of food in their standard weekly shops or purchase smaller amounts more frequently, or whether PLWOw/Ob_with_ or PLWOw/Ob_without_ consume greater quantities of food from sources outside their main weekly shop. Any weight status-related behavioural differences could result in either PLWOw/Ob_with_ or PLWOw/Ob_without_ not providing agreement between purchasing and consumption behaviours, or result in different levels of bias between the two measures, which could have a significant distorting influence on the results obtained in any study if it is not accounted for. Consequently, understanding whether the agreement and bias between purchase and consumption behaviours differ between PLWOw/Ob_with_ and PLWOw/Ob_without_ would add considerable value to the literature.

This study builds upon previous work using STRIDE (Supermarket Transaction Records In Dietary Evaluation) data [4] and aims to establish: (i) whether the introduction of calorific screening thresholds enhances the agreement between estimated consumption (via FFQs) and purchase (via supermarket transaction records) data; and (ii) whether the levels of agreement or bias differ between weight statuses (i.e. PLWOw/Ob_with_ and PLWOw/Ob_without_).

## Methods

### Study design

The present FIO-STRIDE study utilises data from the STRIDE study [4] to evaluate differences in purchase and consumption habits between PLWOw/Ob_with_ and PLWOw/Ob_without_. Calorific screening thresholds were used to remove participants whose purchases were unlikely to represent a substantial proportion of their diet within the study period. Bland-Altman analyses [13] were used to evaluate the agreement and bias between purchase and consumption data, before considering differences by weight status. The original STRIDE study was granted ethical approval by the Social Science Environment and LUBS (AREA) Faculty Research Ethics Committee, University of Leeds on 15 July 2019, with an updated approval on 2nd December 2023 for this follow-on ‘FIO-STRIDE’ study (AREA18-174). Written informed consent was obtained from all participants. All methods were performed in accordance with the relevant guidelines and regulations.

### Overview of original STRIDE study

This section provides a brief overview of the elements of the original STRIDE study relevant to the present FIO-STRIDE study—further details can be found in the original study [4]. Differences in the preprocessing steps between the previous STRIDE study and this FIO-STRIDE study, and the additional analyses considered within the present study are outlined in subsequent sections.

#### Participants

A total of 1 788 participants consented to take part in the original study [4], after approximately 45 000 eligible customers from the retailer’s loyalty card customer database were contacted by the retailer. To be eligible, participants had to be classified as ‘primary shoppers’—defined by the research team in the original STRIDE study, as shoppers who purchased in at least 7 out of 15 food categories on at least 10 occasions in 2019 [4, 14]. This definition was used to exclude customers who only made occasional purchases with the retailer or who only bought specific types of food in the year prior to the study. Participants completed an online questionnaire providing demographic and anthropometric information (date of birth, gender, ethnicity, height, weight and household composition—i.e. the number and ages of other people within their household) and consented to their supermarket transaction records being linked to their weight status.

#### Food frequency questionnaires

Participants completed a validated 170-item semi-quantitative online FFQ from the Scottish Collaborative Group (SCG) [15, 16] to provide details on individual dietary consumption. The FFQ asked the participant to report the frequency (number of days per week) and amounts of each item (number of measures per day) consumed over the previous three months, capturing their usual dietary intake. The 170-items were split into 21 categories: breads; breakfast cereals; milk; cream and yoghurt; cheese; eggs; meats; fish; potatoes, rice and pasta; savoury foods, soups and sauces; vegetables; fruit; puddings; chocolates, sweets, nuts and crisps; biscuits; cakes; spreads and sugar; beverages and soft drinks; alcoholic drinks; other foods and drinks; and vitamin, mineral and food supplements. A previous validation study against an unweighed 7-day food diary reported Spearman correlation coefficients of: 0.37 for energy; 0.48 for fat; 0.58 for saturated fat; 0.47 for protein; and 0.62 for total sugars in 96 adults aged 18–65 years old [16]. Daily nutrient intakes for each participant were estimated from their FFQ by the SCG team as part of their paid FFQ service, using the United Kingdom (UK) National Nutrient Databank [15]. Six nutrients were considered: total energy (Kcal/day); total sugars (g/day); total fat (g/day); total saturated fat (g/day); total protein (g/day) and total sodium (mg/day). These nutrients were chosen to enable comparison with supermarket transaction data using back-of-pack information, as this information is mandatory for products in the UK. For the remainder of the present study, these daily nutrient intakes, calculated from the FFQ, are termed the *estimated individual nutrient intake*.

#### Supermarket transaction data

Household purchases were provided by the retailer in the form of supermarket transaction data from loyalty cards. These data included all food and beverages (including alcoholic beverages) purchased either in store or online with a scanned loyalty card. Household purchased nutrients were estimated from the transaction data by linking products to a product nutrient composition database, based on product data supplied by NIQ Brandbank © 2024 [17], via a unique product code (either the European Article Number (EAN) or Stock-Keeping Unit (SKU)). Mean daily household purchased nutrients were calculated by dividing the total household nutrients purchased by the number of days in the same 3-month timeframe as that covered by the FFQ [4].

Estimated individual purchased nutrients were calculated by proportionally allocating the mean daily household purchased nutrients to the study participant according to UK dietary recommendations for caloric intake by age and gender [10]. As the sex of other household members was unknown, an average of recommended values for males and females was used for those individuals. The study participant was allocated their proportion of the total recommended caloric intake for the household—Table 1 provides an example of this process.

**Table 1:** Example calculation of proportion of mean daily household purchased nutrients allocated to a 30-year-old female living with a 30-year-old partner and a 3-year-old child.

Age	Gender	Recommended caloric intake (Kcal/day)	Proportion allocated to individual
30	Female	1928	$\frac{1928}{(1928+2230+1197.5)}=36 \%$
30	Unknown	2230	
3	Unknown	1197.5	

In this example, the study participant would be allocated 36% of all six of the mean daily household purchased nutrients considered in this study.

The gender of other household members was not collected in this study so the mean recommended caloric intake across males and females was used. Recommended caloric intakes are taken from PHE guidelines [10].

These *estimated individual purchased nutrients* were provided as absolute daily amounts for the individual participating in the study to allow for comparison with the estimated individual nutrient intake (provided by the FFQ).

### Preprocessing differences in the present FIO-STRIDE study

#### Estimation of weight status

To allow comparisons to be made between weight statuses in the present FIO-STRIDE study, participants were grouped according to body mass index (BMI), calculated using self-reported height and weight. Participants were classified as PLWOw/Ob_with_ (BMI ≥ 25) or PLWOw/Ob_without_ (BMI < 25).

#### Screening thresholds

Calorific screening thresholds were used to include/remove customers based on the quantity of food and drinks purchased within the 3-month study period. These thresholds aimed to remove participants whose estimated purchases could not realistically represent their total consumption. The screening thresholds used the estimated individual purchased nutrient values, which only required supermarket transaction data and household composition information. The estimated individual purchased calories value was used for the screening thresholds as it provided a global view of the quantity of household purchases estimated to be purchased for the individual. Since estimates are subject to error (e.g. from household members consuming different proportions of food than the recommended guidelines and from purchases outside of the supermarket or made without the loyalty card), six screening thresholds were considered using estimated individual purchased calories values of: >0 Kcal/day (*n* = 642); ≥500 Kcal/day (*n* = 435); ≥1000 Kcal/day (*n* = 299); ≥1500 Kcal/day (*n* = 184); ≥2000 Kcal/day (*n* = 108) and ≥2500 Kcal/day (*n* = 49).

### Statistical analyses

Following the removal of participants who did not complete FFQs (*n* = 963), had estimated daily energy intake from the FFQ > 8000 Kcal/day (*n* = 2), made no purchases with the retailer in the same 3-month timeframe as the FFQ (*n* = 137) or did not provide heights and/or weights for BMI to be calculated (*n* = 44), a final sample size of 642 participants was present for this study. Bland-Altman analyses were used to assess the agreement and bias between estimated individual nutrient intake (from FFQ data) and estimated individual purchased nutrients (from supermarket transaction data), as this is the standard method for comparing methods in clinical research [13]. Supermarket transaction data were used as the reference value, framing the research question as “*how much is an individual estimated to consume relative to their estimated purchases?*”.

Prior to analysis, daily nutrient values were log-transformed so proportional differences were considered. Two Bland-Altman analyses were performed at each of the six calorific screening thresholds (i.e. using only data from the participants who exceeded the screening thresholds) for each of the six nutrients considered in this study (total energy (Kcal/day); total sugars (g/day); total fat (g/day); total saturated fat (g/day); total protein (g/day) and total sodium (mg/day)). The first assessed the agreement and bias between estimated individual nutrient intake and estimated individual purchased nutrients across the six screening thresholds without comparing by weight status. The second included weight status as a covariate within the modelling process to evaluate differences in agreement and bias across each nutrient between PLWOw/Ob_with_ and PLWOw/Ob_without_.

#### Agreement and bias

Two measures were taken from the Bland-Altman analyses: *agreement* and *bias*. Agreement refers to the consistency of mean differences across a range of values. As data in this study were log transformed, agreement was reached if the proportional differences were the same across a range of nutrient values. This means that agreement was reached if, for example, on average, an individual consumed 10% more, or less, than they were estimated to have purchased across a range of values (e.g. consistently from 500 Kcal/day purchased (absolute difference 50 Kcal/day) up to 2500 Kcal/day purchased (absolute difference (250 Kcal/day)). Agreement between estimated individual nutrient intake and estimated individual purchased nutrients was deemed to have been achieved statistically if the 95% confidence intervals of the regression coefficient for the mean value did not cross zero. Visually, agreement was seen if the regression line was horizontal or near horizontal.

Bias is the mean difference between what is consumed (FFQ) and what is purchased (supermarket transaction data). In this study, this is the proportional difference between estimated individual nutrient intake and estimated individual purchased nutrients (e.g. individuals were estimated to consume 10% more or less of a nutrient than they were estimated to have purchased). Two types of bias are provided: the back-transformed mean of the raw log-transformed differences and the modelled bias at the average nutrient values. The back-transformed mean of the raw log-transformed differences assumes agreement is present (i.e. it will always be horizontal), whereas the modelled version provides the bias and additionally shows whether agreement is present (i.e. it will not always be horizontal). It is important to note that bias can be present even if agreement is reached. For example, the data could show that there is a 20% difference between estimated individual nutrient intake and estimated individual purchased nutrients (i.e. there is bias in the data), but this difference may be consistent across a range of nutrient intakes (i.e. there is agreement in the data). Where this happens, bias is thought to provide insights into purchase and consumption behaviours, since the differences are consistent across a range of nutrient intakes.

To provide further insight into the differences in purchase and consumption behaviours between weight statuses, a mean expected individual consumption value was calculated for each nutrient for PLWOw/Ob_with_ and PLWOw/Ob_without_. This allowed differences in bias and the quantity of food purchased to be considered together in ‘real terms’ between weight statuses. Mean expected individual consumption values were calculated by multiplying the mean estimated individual purchased nutrient value by the modelled bias value for the weight status group. Differences between weight statuses were deemed to be statistically clear when the confidence intervals of the expected consumption values between groups did not overlap.

Data collation, preprocessing and analysis were handled in the LASER secure data environment at the University of Leeds [18]. All analyses were conducted using the *lm()* function in R (v4.3.0).

## Results

### Participant characteristics

Supplementary Tables 1 and 2 provide characteristics for the participants and households included within the analyses. Age and household sizes were similar between weight statuses, but there was a slightly greater proportion of females in PLWOw/Ob_without_ than PLWOw/Ob_with_.

### Impact of calorific screening thresholds on agreement

**Fig. 1:**
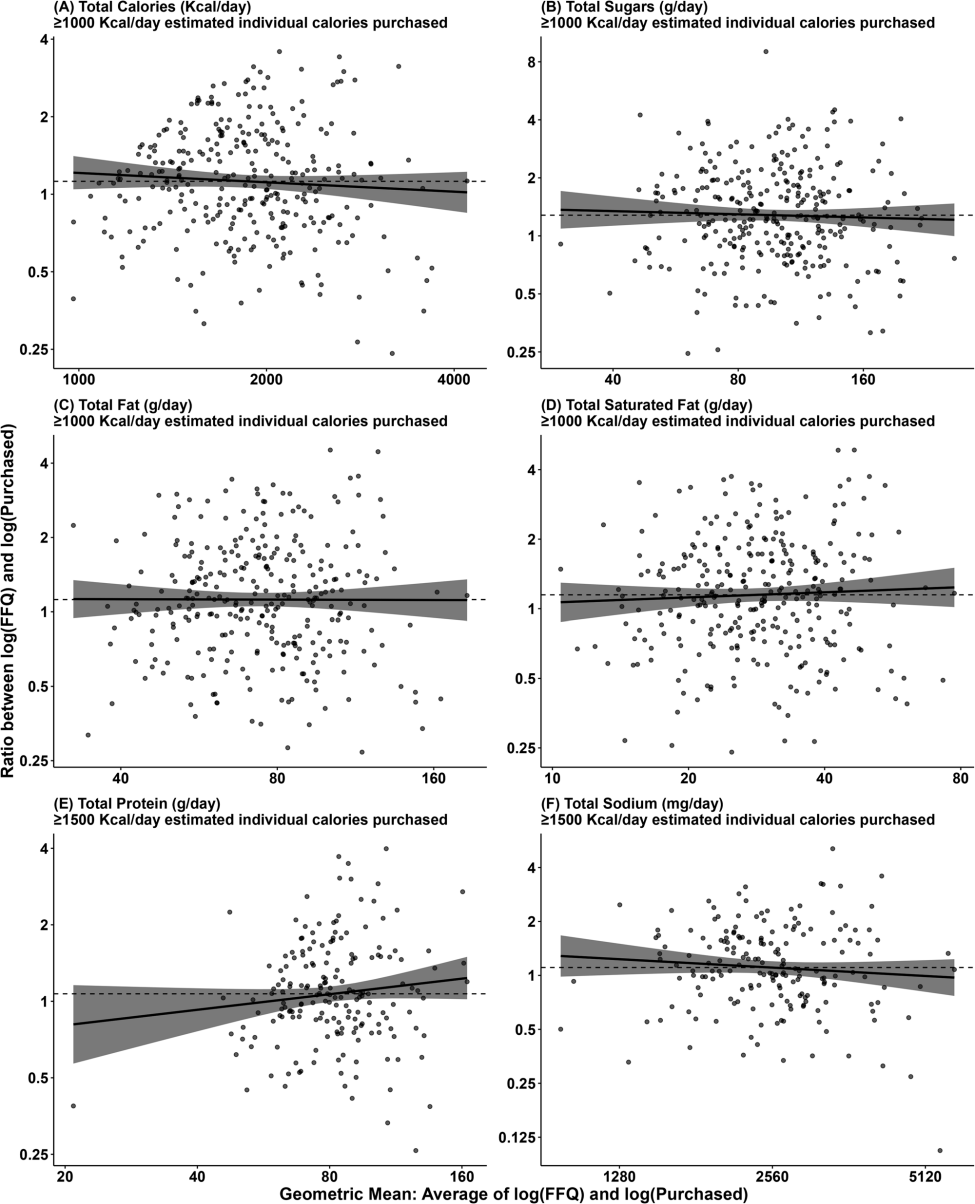
Bland-Altman analyses at the lowest calorific screening threshold where agreement was identified between estimated individual nutrient intake (FFQ) and estimated individual purchased nutrients (supermarket transaction data). **A** Energy, **B**, sugars, **C** fat, **D** saturated fat, **E** protein and **F** sodium. Solid regression line represents modelled bias, with shaded 95% confidence intervals; dashed line represents unmodelled bias. Dots represent individual data points; darker dots represent multiple data points in the same location. An agreement was reached if the regression line was horizontal or near horizontal and the 95% confidence interval contained the *x*-axis.

Figure 1 provides the Bland-Altman plots at the lowest screening threshold whereby agreement was found to be present between estimated individual nutrient intake (FFQ) and estimated individual purchased nutrients (supermarket transaction data) in this study. Agreement was established for energy, sugar, total fat, and saturated fat at screening thresholds of ≥1000 Kcal/day, and for protein and sodium agreement was established at screening thresholds of ≥1500 Kcal/day, ≥2000 Kcal/day, and ≥2500 Kcal/day. Supplementary Figs. 1–6 provide the Bland-Altman plots for each individual nutrient at all screening thresholds.

### Differences between weight statuses

**Fig. 2:**
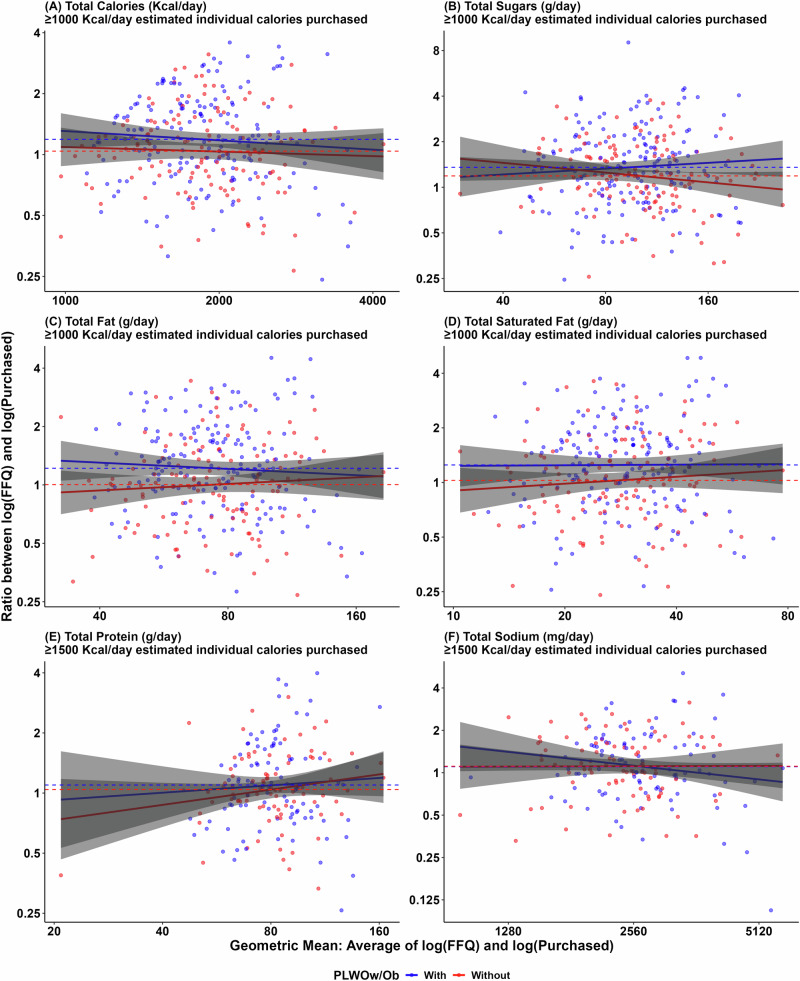
Bland-Altman analyses comparing PLWOw/Ob_with_ (blue) with PLWOw/Ob_without_ (red) at the lowest calorific screening threshold where agreement was identified between estimated individual nutrient intake (FFQ) and estimated individual purchased nutrients (supermarket transaction data). **A** Energy, **B**, sugars, **C** fat, **D** saturated fat, **E** protein and **F** sodium. Solid regression line represents modelled biases, with shaded 95% confidence intervals; dashed line represents unmodelled bias. Dots represent individual data points; darker dots represent multiple data points in the same location. An agreement was reached if the regression line was horizontal or near horizontal and the 95% CIs contained the *x*-axis.

Figure 2 depicts the Bland-Altman plots comparing weight statuses at the lowest screening threshold, where agreement was found between estimated individual nutrient intake and estimated individual purchased nutrients for all data. The data plotted are the same as Fig. 1, except the data are now grouped, with individual regression lines and data points coloured by weight status. No clear differences in agreement were identified between PLWOw/Ob_with_ and PLWOw/Ob_without_.

Table 2 provides the mean estimated individual purchased nutrients for PLWOw/Ob_with_ and PLWOw/Ob_without_, the modelled biases at these values, and the mean expected individual consumption, which combines the expected individual purchased nutrients and estimated biases to provide expected individual consumptions in ‘real terms’. PLWOw/Ob_with_ consumed 19% more energy (95% CI: 9–28%), 36% more sugar (95% CI: 24–48%), 22% more total fat (95% CI: 12–32%), and 25% more saturated fat (95% CI: 15–36%) than they were estimated to have purchased at the retailer. Conversely, PLWOw/Ob_without_ only consumed 19% more sugar (95% CI: 8–31%) than they were estimated to have purchased at the retailer.

**Table 2:** Bias and expected individual consumption at the average estimated individual purchased nutrients for each weight status using the lowest screening threshold Bland-Altman analysis where agreement was found (≥1000 Kcal/day for energy, sugar, fat and saturated fat, ≥1500 Kcal/day for protein and sodium).

	PLWOw/Ob_with_			PLWOw/Ob_without_		
Nutrient	Estimated individual purchased nutrients	Bias	Expected individual consumption	Estimated individual purchased nutrients	Bias	Expected individual consumption
Energy (Kcal/day)	1851	1.19 (1.09–1.28)	2202 (2042–2374)	1871	1.04 (0.95–1.13)	1945 (1784–2121)
Sugar (g/day)	94	1.36 (1.24–1.48)	128 (117–139)	101	1.19 (1.08–1.31)	120 (109–133)
Total fat (g/day)	74	1.22 (1.12–1.32)	91 (84–98)	74	1.00 (0.91–1.10)	74 (68–82)
Saturated fat (g/day)	29	1.25 (1.15–1.36)	36 (33–39)	28	1.03 (0.93–1.13)	29 (26–32)
Protein (g/day)	83	1.10 (1.00–1.21)	91 (82–100)	81	1.04 (0.94–1.15)	84 (76–93)
Sodium (mg/day)	2695	1.10 (0.99–1.23)	2974 (2658–3328)	2376	1.11 (0.99–1.25)	2640 (2347–2969)

Bias represents the proportional difference estimated individual consumption and estimated individual purchased nutrients. Expected individual consumption provides an individual consumption value in absolute terms by multiplying the bias by the estimated individual purchased nutrient. For example, for PLWOw/Ob_with_, the estimated individual purchased calories amount is 1851 which, multiplied by the bias of 1.19, gives an expected individual consumption of 2202 Kcal/day.

Data are provided as mean (95% confidence intervals).

When comparing PLWOw/Ob_with_ vs PLWOw/Ob_without_ in absolute expected individual consumption terms, PLWOw/Ob_with_ had greater expected total fat consumed (91 (95% CI: 84–98) g/day vs. 74 (95% CI: 68–82) g/day) and greater expected saturated fat consumed (36 (95% CI: 33–39) g/day vs. 29 (95% CI: 26–32) g/day). Although the CIs overlapped for all other nutrients, meaning that differences were statistically unclear, some sizeable mean differences were present (e.g. PLWOw/Ob_with_ consumed 257 Kcal/day more energy and 334 mg/day more sodium than PLWOw/Ob_without_).

## Discussion

This study investigated two key objectives: (i) whether the introduction of calorific screening thresholds enhanced the agreement between estimated consumption (estimated individual nutrient intake via FFQs) and purchase (estimated individual purchased nutrients via supermarket transaction records) data; and (ii) whether the level of agreement or bias differed between weight statuses (i.e. PLWOw/Ob_with_ and PLWOw/Ob_without_). Using a novel methodology, which proportionally allocated the total recommended intake for the household to the individual cardholder [4], the results showed that agreement was found between estimated individual nutrient intake and estimated individual purchased nutrients when calorific screening thresholds were employed (≥1000 Kcal/day for energy, sugar, fat and saturated fat; ≥1500 Kcal/day, ≥2000 Kcal/day, and ≥2500 Kcal/day for protein and sodium). There were no differences in agreement between weight statuses, but differences in bias were identified. PLWOw/Ob_with_ were estimated to consume more energy, sugars, total fat and saturated fat than they were estimated to have purchased, whereas PLWOw/Ob_without_ were only estimated to have consumed more sugars.

### Application of screening thresholds

The results of this study indicate that screening participants by estimated individual purchased energy thresholds may improve the correspondence between self-reported dietary intake and objective consumer purchase data. At a threshold of ≥1000 Kcal/day, consistent proportional agreement was achieved for energy, sugar, total fat, and saturated fat, while for protein and sodium higher calorific purchase volumes were needed to observe agreement (≥1500 Kcal/day, ≥2000 Kcal/day, and ≥2500 Kcal/day), potentially reflecting distinct purchasing and reporting patterns for these nutrients. The screening thresholds work by filtering out shoppers who purchase unrealistic quantities of their overall consumption, allowing agreement to be reached between estimated individual nutrient intake (FFQ) and estimated individual purchased nutrients (supermarket transaction data). For protein and sodium, the need for higher thresholds may indicate that these nutrients are subject to greater variability in the location (e.g. meat may be purchased at a butcher’s shop) or frequency (e.g. salt, as a store cupboard ingredient, is typically purchased less frequently, but in higher quantities) of purchases. Additionally, some of the differences in agreement for sodium may have occurred because it is difficult for FFQs to estimate sodium content in food, particularly home-cooked food where discretionary salt can be used during meal preparations and/or at the table [19]. However, across all nutrients, the findings of this study suggest that targeted use of screening thresholds may substantially improve the quality of dietary research by allowing large-scale population studies to be conducted using purchase data that are likely to reflect consumption for individuals. This could have enormous benefits when attempting to, for example, analyse policy changes (e.g. the sugar tax), evaluate dietary changes in different groups or establish the benefit of marketing/educational interventions [20]. Furthermore, the results highlight differences between nutrients which may need to be considered in future study designs. For example to get an accurate representation of sugar intake from consumer purchase data, the individual must have purchased a minimum of 1000 Kcal/day, on average, from the recorded transactions, whereas for protein, they must have purchased a minimum of 1500 Kcal/day.

The application of a screening threshold provides a valuable and effective preprocessing step to establish study participants whose supermarket transaction data is likely to represent a realistic proportion of their diet. However, while it requires relatively little additional information (only household composition and ages are required alongside the supermarket transaction data), these data are currently not widely available within supermarket transaction data studies. It is recommended that researchers, retailers, and third parties who provide nutritional information, for example Composition of Food Integrated Dataset [21], work together to identify methods through which these data can be obtained (e.g. data linkages from surveys or longitudinal data) or estimated (e.g. training machine learning algorithms) to improve future research. Alongside this requirement for additional information, researchers should be aware that the use of screening thresholds invokes additional selection bias within the sample, resulting in two levels of selection bias: 1) the bias invoked by only selecting “primary/diverse” shoppers (confirmed by the participant characteristics in Supplementary Table 1 matching previously shown demographic biases in loyalty cardholders [22]); and 2) the bias invoked by only selecting those shoppers who purchased amounts greater than the screening threshold. If studies wish to accurately understand population level purchasing habits or obtain any population average causal insights using retail transaction data, they must address this selection bias challenge in future research. To date, relatively few studies have used consumer purchase data, which may explain why no study, to the authors’ knowledge, has considered this problem in detail. A further limitation when using screening thresholds for transaction data is the caveat that the screening thresholds identified may be specific to the transaction data considered. The narrow window of the screening thresholds found to be effective ( ≥ 1000 Kcal/day identified agreement for four of the nutrients, with a higher threshold needed for two other ingredients) only adds to this possibility. It is unclear from this study whether these differences have a meaningful, generalisable explanation or occur by chance. Future research should consider this and investigate different screening thresholds within different populations to evaluate whether the screening threshold approach has generalisable utility across different contexts.

### Weight status differences

The discrepancy between estimated individual nutrient intake and estimated individual purchased nutrients observed among PLWOw/Ob_with_ versus PLWOw/Ob_without_ is supported by well-documented discrepancies in dietary self-reporting between the two weight statuses [12]. In absolute terms, PLWOw/Ob_with_ were estimated to consume 19% more energy, 36% more sugars, 22% more total fat, and 25% more saturated fat than they were estimated to have purchased at the retailer, whereas PLWOw/Ob_without_ were only estimated to consume more sugars (19% more). This pattern may reflect a combination of factors, including: (i) PLWOw/Ob_with_ purchased more foods outside of the retailer; (ii) greater amounts of food were consumed having been purchased outside of the 3-month study period; (iii) the estimated individual purchased nutrient value being incorrect (i.e. PLWOw/Ob_with_ may have consumed a greater proportion of the household purchased nutrients than was estimated from their supermarket transaction data); and (iv) the validity of the FFQ in reflecting consumption may differ between groups, for example prior literature suggests that PLOw/Ob_with_ are more likely to under report food intakes when using FFQs [11, 12]. Interestingly, when the mean estimated individual purchased quantities of nutrients and the modelled bias information was combined, only fat and saturated fat showed clear differences by weight status in absolute expected daily consumption. Alone, these differences are somewhat surprising, but it should be noted that unclear (i.e. the confidence intervals overlapped) and reasonably large differences in energy (~250 Kcal/day) and sodium (~300 mg/day) were also observed within the data. More research is required to fully understand these differences within the complexities of the food environment, where the physical, economic, social, and cultural contexts in which people engage with food (including factors such as accessibility, affordability, marketing, and availability) all interact to influence dietary behaviours and health outcomes.

### Limitations

Although this study provides valuable insights to the literature, it is not without its limitations. The first of which being the inclusion of only one retailer, which limits the generalisability of the findings. Future research should attempt to include multiple retailers to gain a more complete picture of purchasing patterns, although it is acknowledged that there are significant logistical challenges to doing so, including the requirement to remove participant anonymity to link retailer purchases. Along similar lines, no information was available regarding whether there were multiple cardholders within a single household, so it is possible that some study participants were lost due to their estimated individual purchased nutrients not meeting a screening threshold, when they may have done if multiple cardholder data were linked together. A second limitation is the use of FFQs for self-reported dietary intake, which are subject to bias and included only 170 items, thereby overlooking other items individuals may purchase. In this study, the term ‘estimated individual nutrient intake’ was specifically used to highlight that FFQs are also an estimation rather than ground truth consumption values. The use of BMI to estimate weight status could also be considered a limitation. As a composite variable [19], BMI is heavily influenced by height but can also be influenced by muscle mass as well as body fat. Consequently, it does have its limitations with respect to categorising PLWOw/Ob_with_ and PLWOw/Ob_without_. Finally, the study is unable to confirm nor disprove hypothetical explanations for differences between self-reported dietary intake and objective purchase data between weight statuses. The explanations provided in this study for these differences should therefore be treated cautiously until supported by further evidence.

## Conclusion

The results of this study show that the application of a calorific screening threshold may be useful as a filtering mechanism for shoppers with inconsistent shopping habits when analysing consumer purchase data. The screening thresholds ensure that estimated individual nutrient intakes can be used to identify differences in purchase and consumption patterns between subgroups of data – for example, by weight status. However, it is important to recognise that the use of these filters requires household information, which is not currently widely available. The screening threshold of ≥1000 Kcal/day estimated individual purchased calories provided agreement for energy, sugars, fat and saturated fat, while the screening threshold of ≥1500 Kcal/day estimated individual purchased calories provided agreement for protein and sodium. There were no differences in agreement between weight statuses, but differences in bias were present. PLWOw/Ob_with_ were estimated to consume greater energy, sugar, fat, and saturated fat than they purchased at the retailer, whereas PLWOw/Ob_wihout_ only consumed greater sugar. These results show that useful insights into individual consumption patterns can be derived from consumer purchase data. However, for the analyses to be understood and interpreted robustly for a general population, there remains considerable methodological work to be done, particularly surrounding the selection biases induced by the recruitment criteria and screening thresholds used in this study.

## Supplementary information

Supplementary Data

## Data Availability

The data that support the findings of this study are held in the Leeds Analytic Secure Environment for Research at the Leeds Institute for Data Analytics, in line with the study participant consent and to protect commercial sensitivity of supermarket transaction data. Restrictions apply to the availability of these data which are not publicly available.
