# Supplementary material for: The impact of calorific screening thresholds and weight status when validating UK supermarket transaction records in dietary evaluation: FIO-STRIDE
Source: Int J Obes (Lond). 2026 May 25;50(8):1791–8. doi: 10.1038/s41366-026-02107-1 (PMC13461337; doi:10.1038/s41366-026-02107-1)

SUPPLEMENTARY DATA

Table S1: Participant characteristics for the 642 participants used in this study. Gender characteristics and participant age groups are count [percentage of individuals within the BMI Group]; other characteristics are mean (SD).

Characteristic	PLWOw/Ob$_{with}$ (*n* = 360)	PLWOw/Ob$_{without}$ (*n* = 282)
**Male**	110 [30.5%]	67 [23.8%]
**Female**	250 [69.5%]	215 [76.2%]
**Age Group 18-24**	0 [0.0%]	6 [2.1%]
**Age Group 25-34**	19 [5.3%]	19 [6.7%]
**Age Group 35-44**	41 [11.4%]	39 [13.8%]
**Age Group 45-54**	73 [20.3%]	61 [21.6%]
**Age Group 55-64**	121 [33.6%]	72 [25.5%]
**Age Group 65-74**	89 [24.7%]	72 [25.5%]
**Age Group 75+**	17 [4.7%]	13 [4.6%]
**Mean Age**	56.9 (11.9)	55.1 (14.1)
**Mean BMI**	31.5 (6.65)	22.0 (2.15)

Table S2: Participant age for the 642 participants used in this study. Household size is mean (SD); other descriptives are presented as count [percentage of individuals within the BMI Group]. Adults defined as 18 years or older, Children defined as younger than 18 years old.

Household Characteristics	$PLWOw/Ob_{with}$ (n = 360)	$PLWOw/Ob_{without}$ (n = 282)
**Household Size**	2.18 (1.09)	2.24 (1.13)
**1 Person Households**		
**1 Adult**	91 [25.3%]	66 [23.4%]
**2 People Households**		
**2 Adults**	164 [45.6%]	134 [47.5%]
**1 Adult, 1 Child**	5 [1.4%]	4 [1.4%]
**3 People Households**		
**3 Adults**	26 [7.2%]	22 [7.8%]
**2 Adults, 1 Child**	24 [6.7%]	10 [3.5%]
**1 Adult, 2 Children**	5 [1.4%]	3 [1.1%]
**4 People Households**		
**4 Adults**	11 [3.1%]	6 [2.1%]
**3 Adults, 1 Child**	4 [1.1%]	7 [2.5%]
**2 Adults, 2 Children**	15 [4.2%]	19 [6.7%]
**1 Adult, 3 Children**	1 [0.3%]	1 [0.4%]
**5 People Households**		
**5 Adults**	1 [0.3%]	1 [0.4%]
**4 Adults, 1 Child**	3 [0.8%]	2 [0.7%]
**3 Adults, 2 Children**	3 [0.8%]	1 [0.4%]
**2 Adults, 3 Children**	4 [1.1%]	2 [0.7%]
**6+ People Households**		
**6 Adults**	0 [0.0%]	1 [0.4%]
**5 Adults, 1 Child**	1 [0.3%]	0 [0.0%]
**4 Adults, 2 Children**	2 [0.6%]	0 [0.0%]
**2 Adults, 4 Children**	0 [0.0%]	1 [0.4%]
**7 Adults**	0 [0.0%]	2 [0.7%]

Figure S1: Bland-Altman analyses for total calories (Kcal/day) showing agreement and bias at the six calorific screening thresholds considered in this study. Solid regression line represents modelled bias, with shaded 95% confidence intervals; dashed line represents unmodelled bias. Dots represent individual data points; darker dots represent multiple data points in

the same location. Agreement was reached if the regression line was horizontal or near horizontal and the 95% confidence interval contained the x-axis.

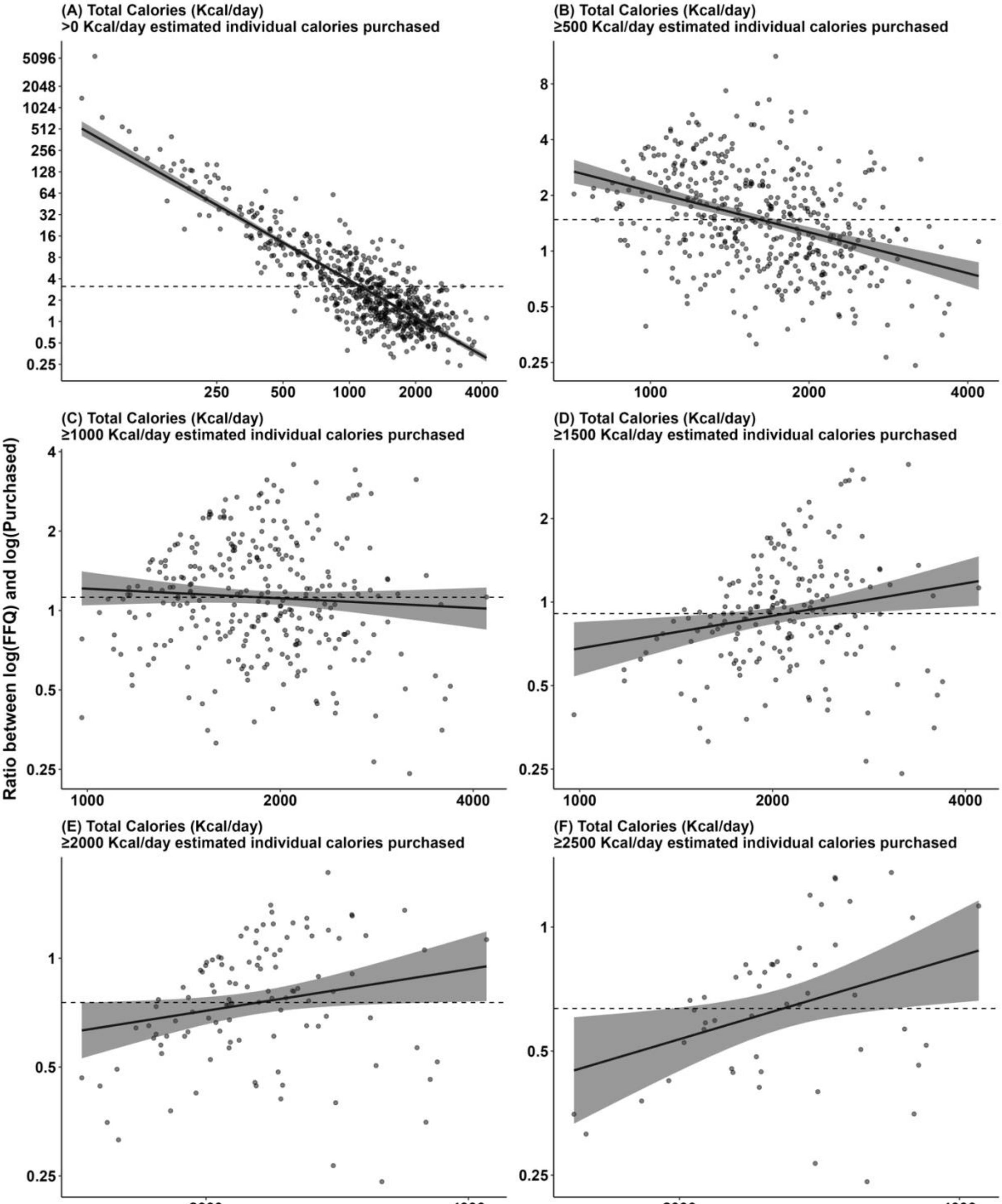

Figure S2: Bland-Altman analyses for total sugars (g/day) showing agreement and bias at the six calorific screening thresholds considered in this study. Solid regression line represents modelled bias, with shaded 95% confidence intervals; dashed line represents unmodelled bias. Dots represent individual data points; darker dots represent multiple data points in the same location. Agreement was reached if the regression line was horizontal or near horizontal and the 95% confidence interval contained the x-axis.

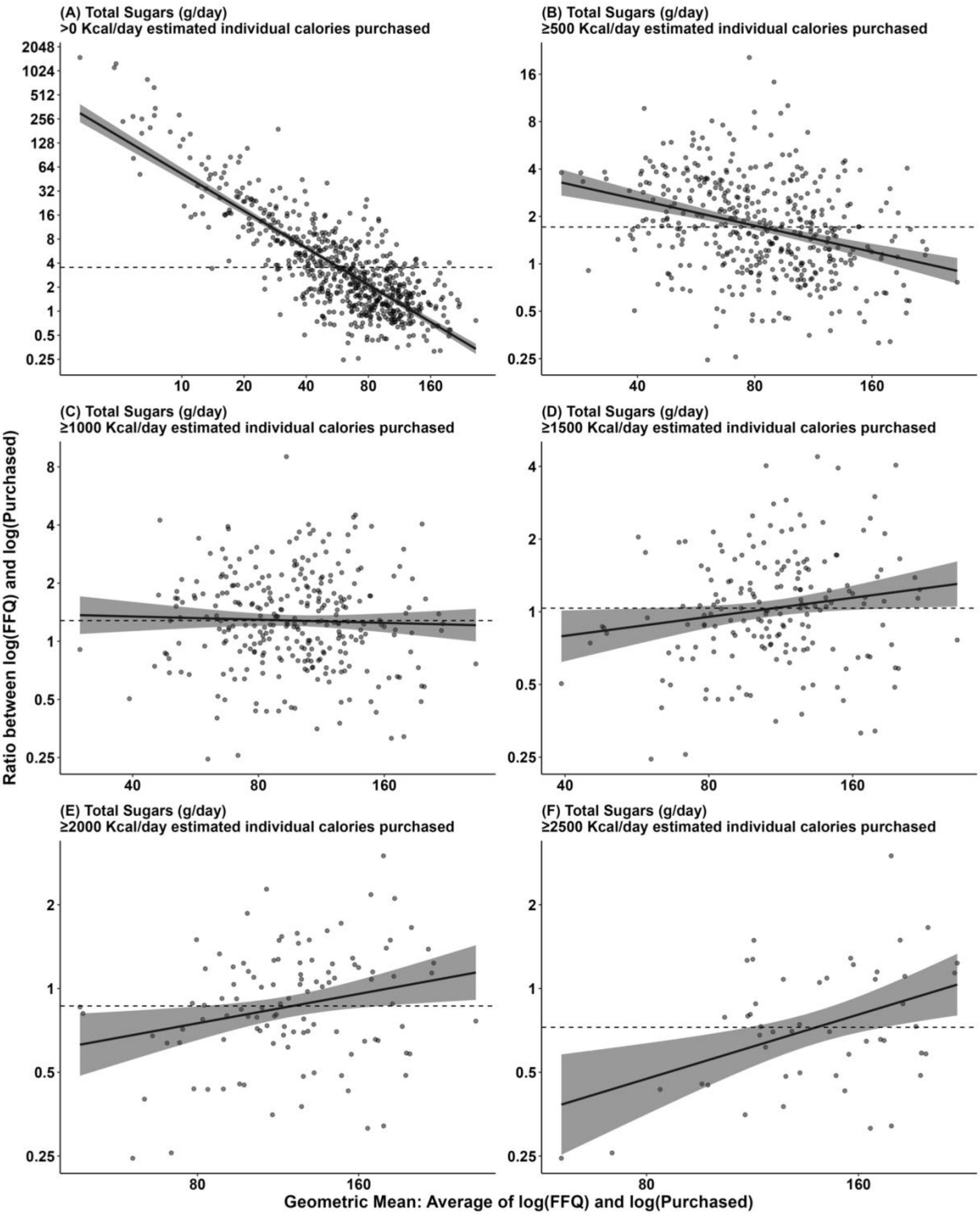

Figure S3: Bland-Altman analyses for total fat (g/day) showing agreement and bias at the six calorific screening thresholds considered in this study. Solid regression line represents modelled bias, with shaded 95% confidence intervals; dashed line represents unmodelled bias. Dots represent individual data points; darker dots represent multiple data points in the same location. Agreement was reached if the regression line was horizontal or near horizontal and the 95% confidence interval contained the x-axis.

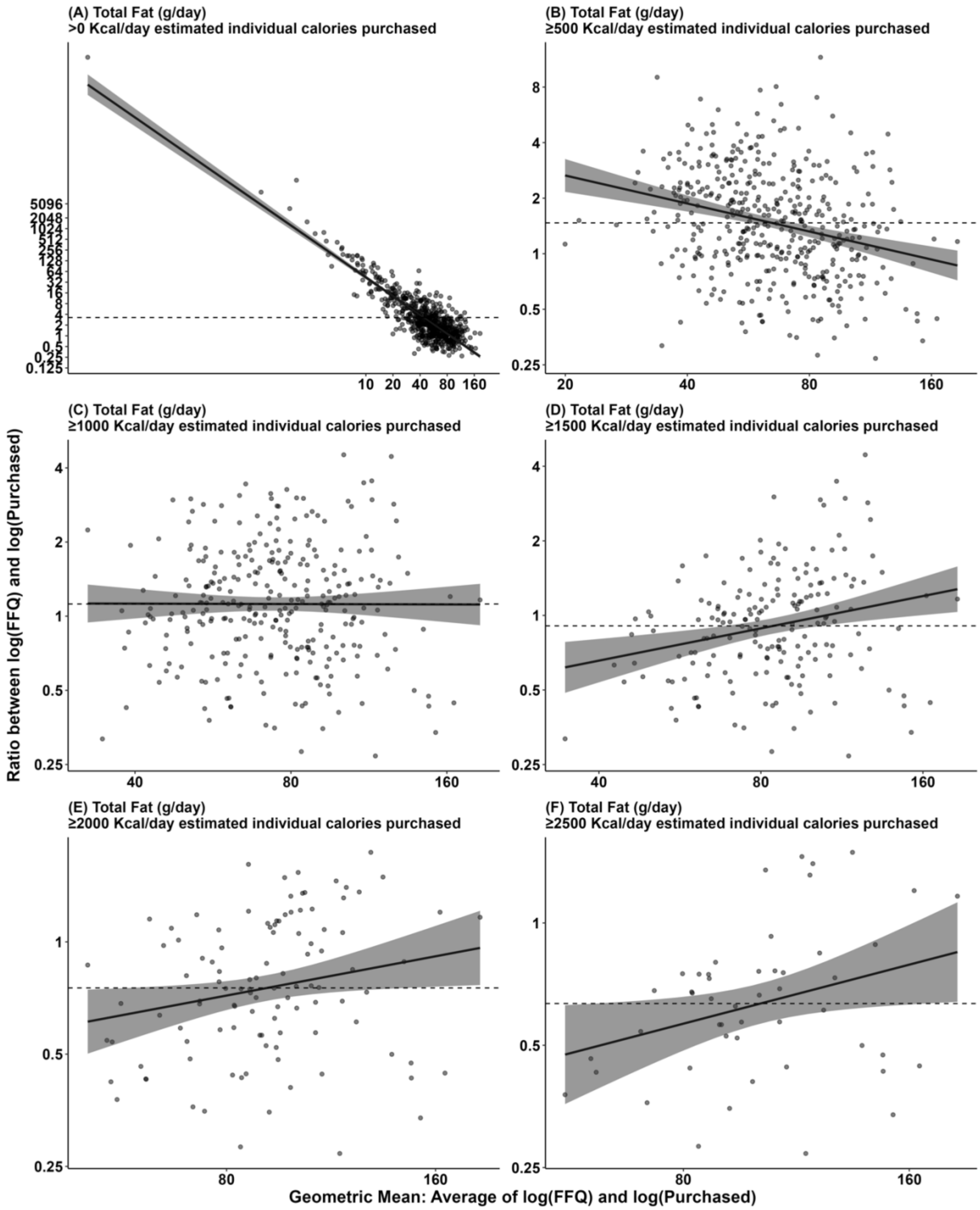

Figure S4: Bland-Altman analyses for total saturated fat (g/day) showing agreement and bias at the six calorific screening thresholds considered in this study. Solid regression line represents modelled bias, with shaded 95% confidence intervals; dashed line represents unmodelled bias. Dots represent individual data points; darker dots represent multiple data points in the same location. Agreement was reached if the regression line was horizontal or near horizontal and the 95% confidence interval contained the x-axis.

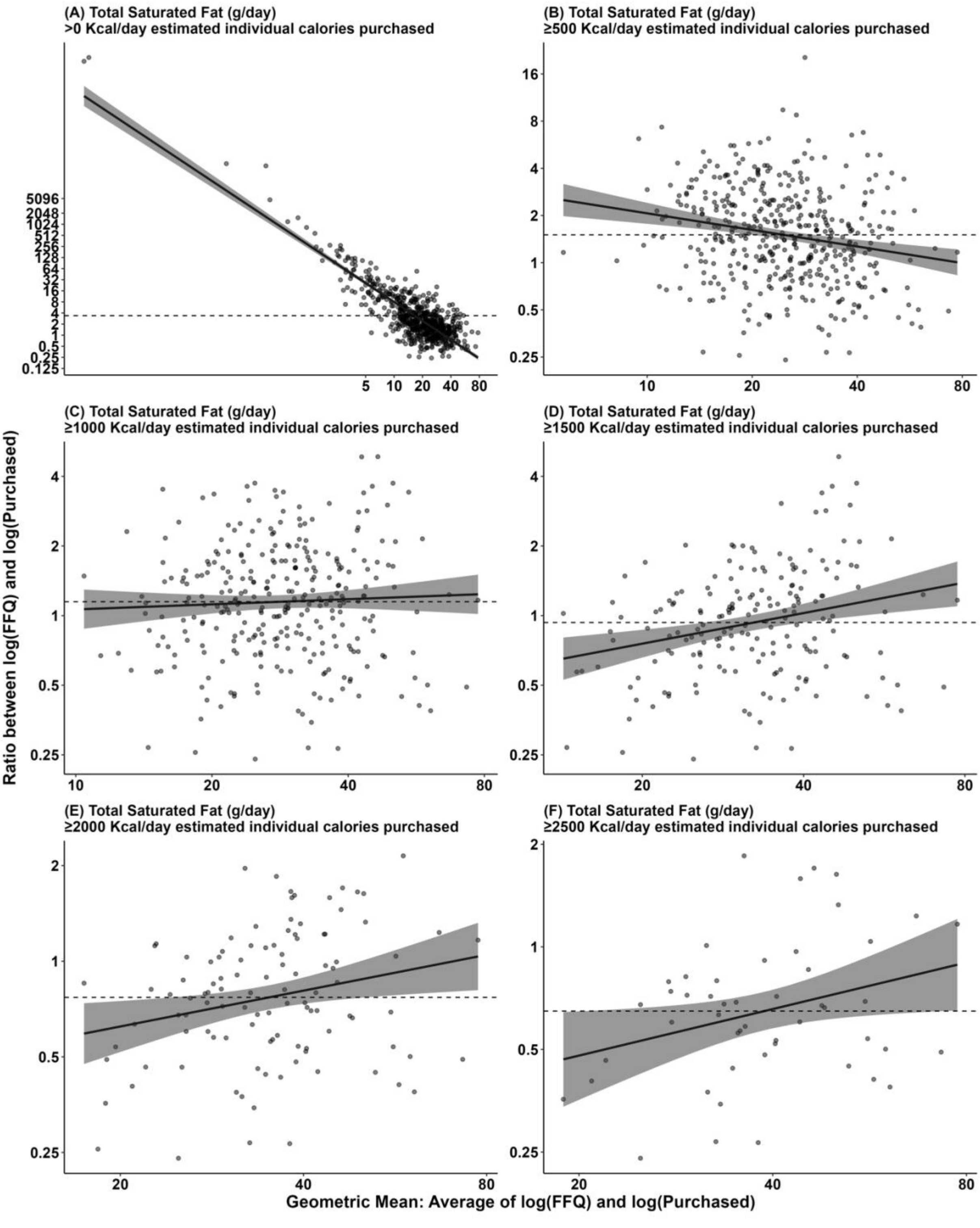

Figure S5: Bland-Altman analyses for total protein (g/day) showing agreement and bias at the six calorific screening thresholds considered in this study. Solid regression line represents modelled bias, with shaded 95% confidence intervals; dashed line represents unmodelled bias. Dots represent individual data points; darker dots represent multiple data points in the same location. Agreement was reached if the regression line was horizontal or near horizontal and the 95% confidence interval contained the x-axis.

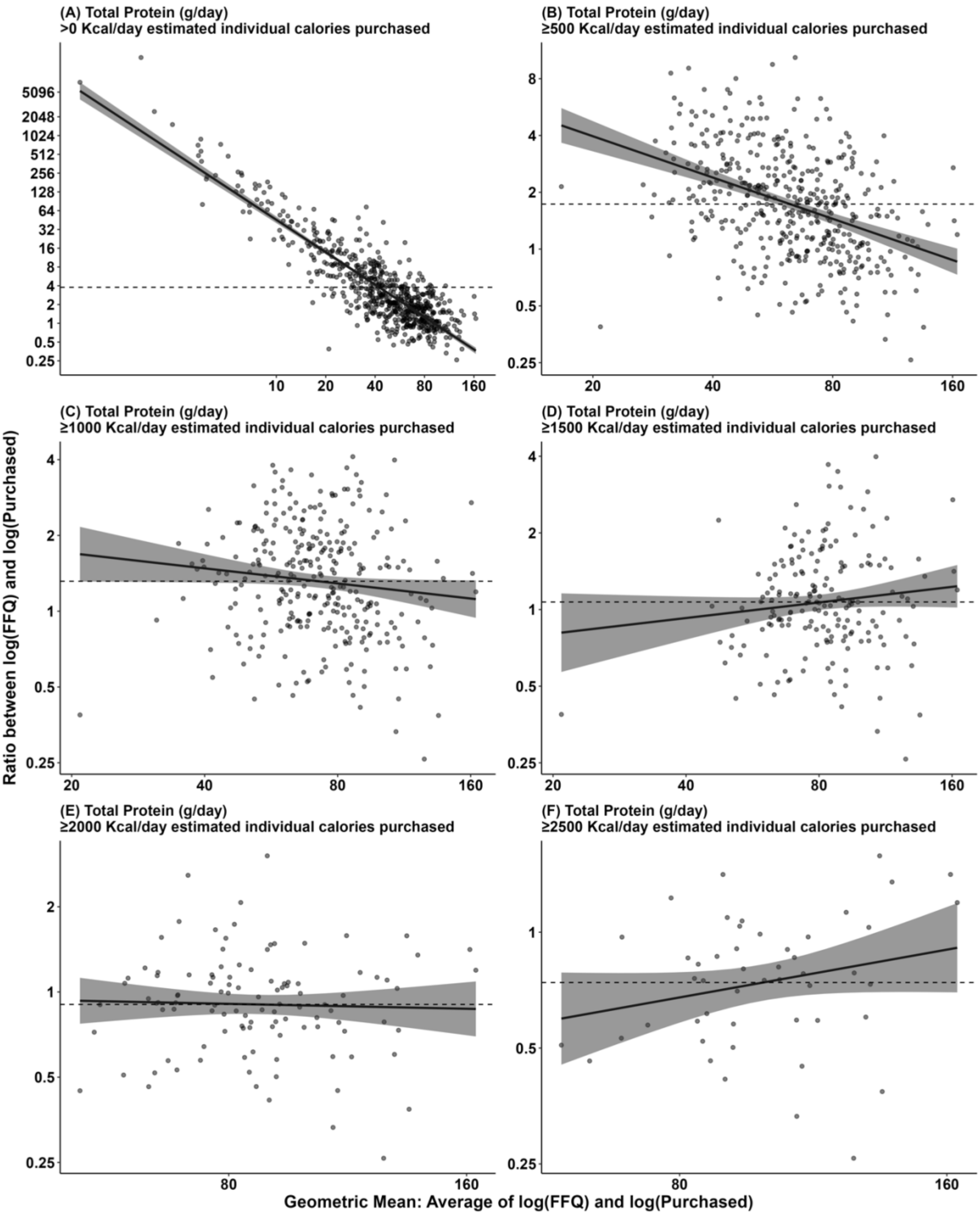

Figure S6: Bland-Altman analyses for total sodium (mg/day) showing agreement and bias at the six calorific screening thresholds considered in this study. Solid regression line represents modelled bias, with shaded 95% confidence intervals; dashed line represents unmodelled bias. Dots represent individual data points; darker dots represent multiple data points in the same location. Agreement was reached if the regression line was horizontal or near horizontal and the 95% confidence interval contained the x-axis.

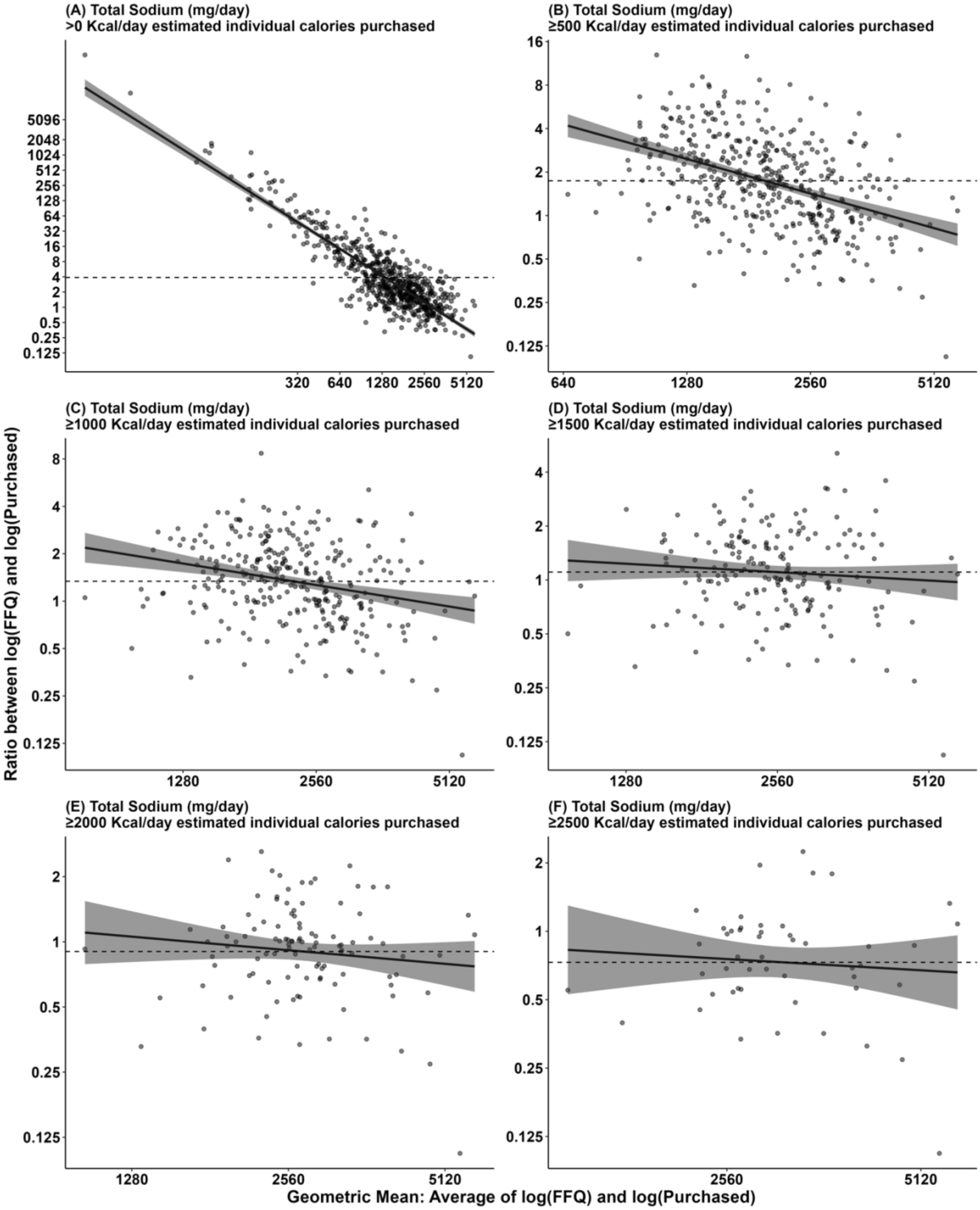

Supplement: Supplementary file 1 [file 41366_2026_2107_MOESM1_ESM.pdf]
